# Molecular Detection of Equine Herpesviruses from Field Outbreaks in Donkeys in Northwest Amhara Region, Ethiopia

**DOI:** 10.1155/2024/9928835

**Published:** 2024-10-01

**Authors:** Anmut Worku, Wassie Molla, Ambaye Kenubih, Haileleul Negussie, Bemrew Admassu, Mebrat Ejo, Gashaw Getaneh Dagnaw, Abebe Belete Bitew, Tewodros Fentahun, Kalkidan Getnet, Haileyesus Dejene, Kassahun Berrie, Saddam Mohammed Ibrahim, Abebe Tesfaye Gessese, Bereket Dessalegn, Mastewal Birhan, Melkie Dagnaw Fenta, Mebrie Zemene Kinde

**Affiliations:** ^1^ Department of Veterinary Pathobiology College of Veterinary Medicine and Animal Sciences University of Gondar, Gondar, Ethiopia; ^2^ Department of Veterinary Epidemiology and Public Health College of Veterinary Medicine and Animal Sciences University of Gondar, Gondar, Ethiopia; ^3^ Department of Clinical Medicine College of Veterinary Medicine and Agriculture Addis Ababa University, Bishoftu, Ethiopia; ^4^ Department of Veterinary Biomedical Sciences College of Veterinary Medicine and Animal Sciences University of Gondar, Gondar, Ethiopia; ^5^ Vaccine and Diagnostics Research & Development Division Armauer Hansen Research Institute (AHRI), Addis Ababa, Ethiopia; ^6^ Department of Clinical Medicine College of Veterinary Medicine and Animal Sciences University of Gondar, Gondar, Ethiopia

## Abstract

Equine herpesviruses pose a threat to equine health and potentially cause substantial economic losses to the global equine industry. EHV outbreaks have been reported in various parts of Ethiopia and the Amhara region specifically. This study aimed to detect EHVs from suspected outbreak cases in selected districts of the Northwest Amhara region. A cross-sectional study was performed from January 2022 to July 2022 to detect EHVs from suspected outbreak cases. Clinical observation was conducted for the presumptive identification of equine herpesvirus infection, and nasopharyngeal swab samples were collected for molecular detection of the viruses for confirmation. Out of 463 donkeys observed, 23 donkeys showed clinical signs suggestive of equine herpesvirus infection. Samples from 10 suspected donkeys were further subjected to polymerase chain reaction (PCR) test, amplifying ORF30 for EHV-1 and gB for EHV-2 and EHV-5. Among the 10 donkeys tested, seven (*n* = 7) were positive for EHV-5. All ten (*n* = 10) tested donkeys were negative for EHV-1 and EHV-2. EHV-5 was detected in animals with nervous signs, respiratory signs, a combination of nervous and respiratory signs, and a combination of abortion, respiratory, and nervous signs. Generally, only EHV-5 was identified from the outbreak, and more detailed epidemiological/molecular studies should be performed to better understand its dynamics and inform preventive measures.

## 1. Introduction

Equine herpesviruses (EHVs) are globally prevalent viruses of equines [1], posing significant threats to equine health and leading to substantial economic losses in the equine industry worldwide [2]. Among EHVs, EHV-1 is known to cause respiratory disease, abortion, neonatal mortality, and paralytic neurological disorders [3–6], while EHV-4 primarily induces respiratory ailments. EHV-4 has also been sporadically linked to cases of abortion [5]. Although the clinical significance of EHV-2 and EHV-5 is not well-defined, reports indicate their association with respiratory diseases [7].

The establishment of latency and subsequent reactivation are crucial in the epidemiology of equine herpesvirus infections. Many infected animals enter a lifelong latent phase, ensuring the persistence of EHVs and providing opportunities for reactivation during periods of stress throughout the animal's life [3, 8]. Reactivation of latent infection can also be induced by corticosteroid drugs and chorionic gonadotrophin (CG) hormone released by the placenta during early pregnancy [3]. Transmission occurs through the inhalation of respiratory secretions from infected animals and direct or indirect contact with contaminated objects, facilitating the easy dissemination of the viruses [3, 6, 8].

EHVs have been reported in some parts of Ethiopia. In the Amhara Region, during the period of 2011–2013, outbreaks of equine neurological problems were reported, and EHV-1 has been isolated from these outbreaks. In these outbreak investigations, only equines that showed clinical manifestations indicative of EHV-1 myeloencephalopathy were included [9]. In addition, EHV-1 and EHV-4 are found to be highly prevalent in North Shewa Zone, at 71.9% [10], and in Central Ethiopia, at 51% [11], via seroprevalence studies. EHV-1, EHV-4, EHV-2, and EHV-5 were also detected in Angolelana Tera, Abichuna Gnea, and South Achefer [12]. Furthermore, Worku et al. [13] reported 65.9% EHV-1/-4 seroprevalence in Northwest Amhara, Ethiopia.

Moreover, recent occurrences of suspected EHV infections presenting with respiratory and neurological problems, as well as cases of abortion, have been frequently reported in the Belesa and Takusa districts of the Northwest Amhara region (personal communication).

Despite the identification of various types of EHVs in certain parts of the country and the occurrence of suspected EHV outbreaks, no previous investigations have been conducted to identify EHVs specifically in the Northwest Amhara Region, except for the isolation of EHV-1 from equines with clinical signs of EHV-1 myeloencephalopathy by Negussie et al. [9]. However, the clinical outcomes and prevalence of EHV infections are influenced by the type of viruses and geographical regions [4]. Therefore, identifying the specific EHVs present in this area is crucial for managing outbreaks effectively as such information can be utilized to develop and implement preventive and control strategies [5, 14]. Hence, this study aimed to detect EHVs in districts of the Northwest Amhara region where EHV outbreaks were suspected.

## 2. Methods and Materials

### 2.1. Study Area, Study Period, and Study Design

**Figure 1:**
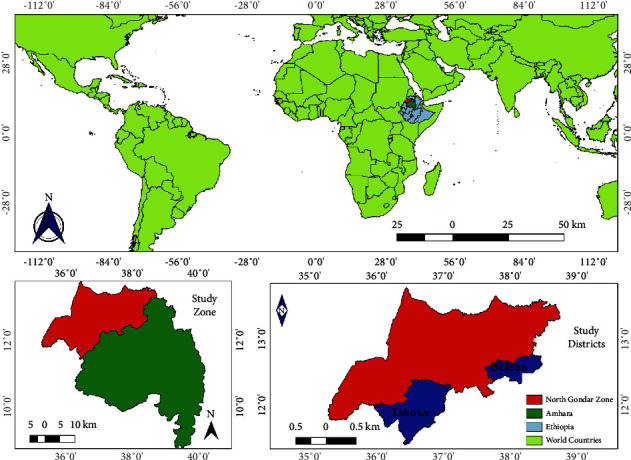
Map that shows the study area. The map is created using QGIS version 3.14.

A cross-sectional study was conducted from January 2022 to July 2022 in two selected districts (namely, Takusa and Belesa) of the Northwest Amhara Region, Ethiopia (Figure 1), where EHV outbreaks in donkeys were suspected. Takusa district is located in the North Gondar Zone of the Amhara Region, about 830 km northwest of Addis Ababa (the capital city of Ethiopia) and 135 km northwest of Bahir Dar (the capital of the Region). It is situated at 12°1ʹ56.64″ N and 36°56ʹ47.76″ E in the northwestern part of Ethiopia, with altitudes ranging from 600 to 2000 meters above sea level (m.a.s.l.). Similarly, Belesa is another district in the North Gondar Zone, located approximately 736 km away from Addis Ababa and 170 km from Bahir Dar. It is situated between 12°19′36.978″N and 12°45′38.019″N latitude and 37°37′10.453″E and 38°28′2.859″E longitude, with altitudes varying from 1496 to 2000 m.a.s.l. [15].

### 2.2. Study Populations and Sample Size

The study population included donkeys dwelling in selected districts (Takusa and Belesa) of the Northwest Amhara Region (Ethiopia) where EHV outbreaks occurred. In these districts, donkeys are the most populous equids, and the outbreak was observed only in donkeys; as a result, horses and mules were not included in the outbreak investigation. Donkeys that showed signs of EHV infection were used for outbreak investigation. Donkeys of both sexes, all age groups, and different body conditions were included. The study animals were categorized into three age groups based on dentition: <3 years old, between 3 and 8 years old, and >8 years old [16]. The body condition of the study animals was also grouped into three categories: poor (BCS 1), moderate (BCS 2), and good (BCS 3), according to NAWC (2005). Out of 463 donkeys observed, 23 donkeys showed clinical signs presumptive of equine herpesvirus infection.

### 2.3. Outbreak Investigation

A field investigation was conducted during the outbreak, involving the clinical investigation of individual cases. Clinical information, including fever (rectal temperature ≥38°C), coughing, lacrimation, nasal discharge, enlargement of the mandibular and/or retropharyngeal lymph nodes, lethargy, anorexia, incoordination of the hind limbs, leaning against a wall or other secure surface, ataxia or wobbly gait, urinary incontinence, and recumbency with an inability to rise, were recorded. The presence of abortion and neonatal death cases was also considered. Appropriate samples were collected from equids showing signs suggestive of EHV infection. A total of 463 donkeys (242 from Takuasa and 221 from Belesa) were observed for equine herpesvirus infection in areas where outbreaks were suspected or reported. Out of the 463 donkeys observed, 23 (12 from Takuasa and 11 from Belesa) showed clinical signs presumptive of equine herpesvirus infection.

### 2.4. Sample Collection

Nasopharyngeal swab samples were collected aseptically from all clinically affected animals (*n* = 23) using sterile swabs for further confirmation of EHVs via molecular tests. The collected swabs were inserted into cryovials containing viral transport media, and the cryovials were properly labeled and immediately placed in an ice box. Afterwards, the samples were transported to the National Veterinary Institute (NVI) (Ethiopia), where they were analyzed. The samples were kept at −70°C until further processing. Due to the scarcity of reagents, only 10 out of the 23 samples collected were processed.

### 2.5. DNA Extraction and Polymerase Chain Reaction (PCR)

Viral DNA was directly extracted from nasal swab samples stored at −70°C using a QIAamp DNA Mini Kit (Qiagen, Hilden, Germany), following the manufacturer's instructions. Due to a shortage of reagents, only 10 samples were randomly selected to be processed by PCR.

Primers specific to EHV-1, EHV-2, and EHV-5 were employed for PCR amplification, as shown in Table 1. PCR was not performed for EHV-4 due to the lack of a primer. A segment of 466 base pairs (bp) of the ORF30 region was targeted for EHV-1 detection, while regions of 444 bp and 283 bp of the glycoprotein B gene (gB) were amplified for the identification of EHV-2 and -5, respectively.

PCR was performed using Agilent's Herculase II fusion DNA polymerase (Agilent Technologies, Inc., Santa Clara, CA, USA). Amplification of DNA was carried out in a total volume of 25 *µ*L PCR reaction mixtures containing 12 *μ*L of RNase-free water, 5 *μ*L Herculase II reaction buffer, 0.5 *μ*L Herculase II fusion DNA polymerase, 0.5 *μ*L of 25 mM of each deoxynucleoside triphosphate (dNTP) mix, 1 *μ*L of each forward and reverse primer, 2.5 *μ*L of dimethyl sulphoxide (DMSO), and 2.5 *μ*L template DNA. In each reaction, a positive control (with DNA template from reference EHV-1, EHV-2, and EHV-5 strains which were obtained from NVI virus stock that had been isolated and sequenced in 2017) and a negative control (nuclease-free water without DNA template) were included.

For the amplification of the ORF30 region, an initial denaturation step at 95°C for 15 minutes was followed by 34 cycles of denaturation at 94°C for 1 minute, annealing at 55.5°C for 1 minute, extension at 72°C for 1 minute, and a final extension at 72°C for 10 minutes. The PCR assays targeting the gB genes of EHV-2 and EHV-5 utilized the following thermocycling conditions: initial denaturation at 95°C for 5 minutes, followed by 40 cycles of amplification with denaturation at 95°C for 30 seconds, annealing at 60°C for 30 seconds, extension at 72°C for 45 seconds, and a final extension at 72°C for 10 minutes.

**Figure 2:**
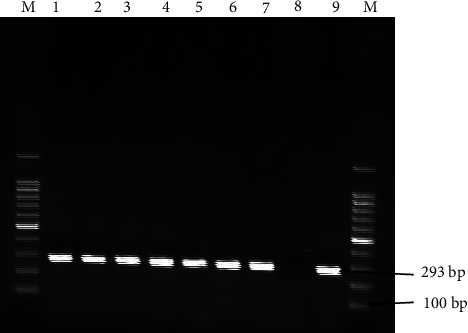
Representative agarose gel electrophoresis of DNA-amplified product generated by targeting specific regions of EHV-5 gB gene (293 bp) on 1.5% agarose gel with a DNA molecular weight marker (MWM) of 100 bp. Lanes 1–7 were positive samples. N, negative control; P, positive control; M, molecular marker.

Detection of the final PCR products was carried out in a 1.5% (w/v) agarose gel prepared with 0.5X Tris-borate-EDTA buffer stained with 4 *μ*L GelRed. Each PCR product (5 *μ*L) was mixed with 6X loading buffer and loaded into separate wells of the preprepared gel, while a 100 bp plus DNA molecular marker was loaded onto the first and last lanes. The gel was run at 120 V for 1 hour and 20 minutes in an electrophoresis chamber. Subsequently, the gels were examined for specific size bands using a UV transilluminator. Bands of 466 bp, 444 bp, and 293 bp were identified as specific to EHV-1, EHV-2, and EHV-5, respectively (Figure 2).

## 3. Results

### 3.1. Clinical Findings

**Figure 3:**
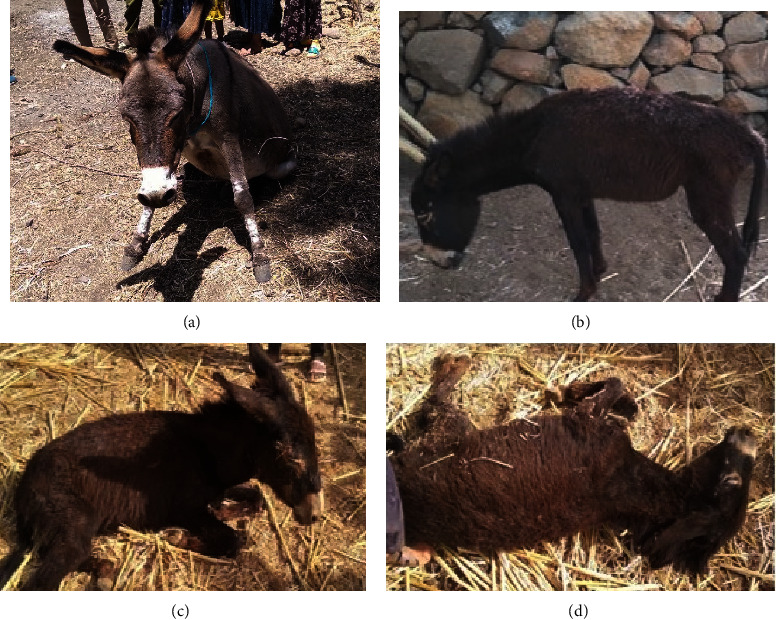
Clinically diseased donkeys suspected of EHV infection. Symptoms include (a) hind limb paralysis or weakness, (b) incoordination and head tilt, (c) lacrimation, and (d) recumbence and inability to rise.

During the period from January 2022 to July 2022, EHV outbreaks in Takusa and Belesa districts of the Northwest Amhara region (Ethiopia) were recorded. Out of 463 donkeys observed, 23 donkeys (12 from Takusa and 11 from Belesa) showed clinical signs presumptive of equine herpesvirus infection. In the outbreak, donkeys were carefully examined for the presence of characteristic clinical signs of EHV. Of the 23 EHV-affected donkeys, 13 (56.52%) had raised rectal temperature, 3 (13.04%) had lacrimation (Figure 3(c)), 11 (47.83%) had nasal discharge, 4 (17.39%) had paralysis (Figure 3(a)), 2 (8.69%) had head tilt (Figure 3(b)), 3 (13.04%) were recumbent and unable to rise (Figure 3(d)), and 5 (21.74%) had ataxia and paresis. Furthermore, abortion was observed in 4 (17.39%) cases (Table 2).

### 3.2. Number of Cases with Different Variables/Factors

Higher percentages of the cases were female donkeys compared to male donkeys. Furthermore, the clinical findings showed that donkeys with poor body condition had the highest percentage of cases, followed by donkeys with moderate body condition. All donkeys with presumptive equine herpesvirus had contact with other equids. Details of clinical findings of the EHV outbreak based on different variables/factors are provided in Table 3.

### 3.3. Polymerase Chain Reaction

Nasal samples from 10 presumably identified donkeys were further subjected to a polymerase chain reaction test, amplifying ORF30 for EHV-1 and gB for EHV-2 and EHV-5. Among the 10 equids tested, seven (*n* = 7) were positive for EHV-5. All ten (*n* = 10) tested equids were negative for EHV-1 and EHV-2. Details of the animals and PCR results for equine herpesvirus types 1, 2, and 5 in nasal swab samples are provided in Table 4. The samples and control PCR amplification bands are indicated in Figure 2, with band sizes of 293 bp specific to EHV-5. EHV-5 was detected in animals with nervous signs, respiratory signs, a combination of nervous and respiratory signs, and a combination of abortion, respiratory, and nervous signs.

## 4. Discussion

Equine herpesviruses are widespread viruses in the equine population worldwide, posing a threat to equine health and causing significant economic losses to the equine industry globally [2]. There are about five EHVs that cause extensive economic losses due to abortion, death and euthanasia, treatment and control costs, and loss of work functions (Reed et al., 2004). The aim of this study was to detect EHVs in equids suspected of EHV infection in districts of the Northwest Amhara region where an EHV infection outbreak was suspected. Clinical and molecular methods were employed for presumptive disease identification and molecular virus detection, respectively. The findings of this study indicated an association of EHV-5 with suspected cases of EHV infection in the study areas. Clinical and molecular methods were employed in the presumptive identification of the disease and molecular detection of the virus, respectively.

EHV-5 was detected in clinically diseased equids exhibiting nervous signs, respiratory signs, a combination of nervous and respiratory signs, and a combination of respiratory, nervous, and abortion symptoms. In line with our findings, Temesgen et al. [20] reported molecular evidence of EHV-1, EHV-2, and EHV-5 infection in donkeys and horses with signs of respiratory disease. They documented that donkeys and horses have varying levels of susceptibility to EHVs, with EHV-1 and EHV-5 detected in higher proportions in donkeys, while EHV-2 was detected in higher proportions in horses. Similarly, Negussie et al. [12] and Wondimagegnehu et al. [21] identified EHV-5 in donkeys with respiratory diseases, although the observed difference was not statistically significant when compared to EHV-5 identified in clinically healthy equids. Additionally, El-Hage et al. [22] reported the association of EHV-5 with equine respiratory disease. However, the findings of our study disagree with the findings of Thompson et al. [23] who suggested that the role of EHV-5 in causing equine diseases is unclear, except in some instances of respiratory disease. This difference may be attributed to variations in equine breed and age [24], as breed-specific differences in susceptibility to EHV-5 infections have been proposed by Stasiak et al. [24]. Additionally, EHV-5 has been implicated in cases of equine multinodular pulmonary fibrosis (EMPF), a nodular fibrotic interstitial pulmonary disease [25], as well as in a few instances of equid abortions. Regardless of the herpesvirus involved, the pathogenesis of abortion is linked to vascular necrosis (Davis, 2018). Similarly, the recovery of EHV-5 from cases of nervous and abortion symptoms in our study may be attributed to vascular inflammation and nervous tissue damage resulting from the reactivation of the latent virus. EHV-5 establishes functional latency in circulating lymphocytes and trigeminal ganglia, from which the virus can be reactivated, leading to the shedding of infectious virus from the host, which can cause clinical disease [24].

Furthermore, differences may arise from variations in the genetic and dynamic characteristics of EHV-5. Genetic variability and the combination of equid gammaherpesvirus strains infecting a horse may have clinical significance [25, 26]. Phylogenetic analyses have revealed genetic heterogeneity among EHV-5 strains [27]. Ethiopian gammaherpesvirus strains also exhibit vast genetic diversity, with identified EHV-5 strains showing similarities ranging from 95.1% to 100% [12]. Similarly, the analysis of Turkish gammaherpesviruses has shown a high degree of genetic heterogeneity among their gB gene nucleotide sequences (Dagalp et al., 2018). The nature and severity of herpesvirus infections are influenced by the type and strain of herpesviruses as well as geographical factors [28].

In our study, EHV-1 and EHV-2 were not detected in any of the tested donkeys (*n* = 10). This disagrees with the previous findings of Negussie et al. [9], Negussie et al. [12], Temesgen et al. [20], Vala et al. [29], and Wondimagegnehu et al. [21], who identified EHV-1 and EHV-2 in cases of respiratory and nervous problems. Tong et al. [30] also isolated and identified EHV-1 as an etiological agent of abortions in donkeys. Differences in sample size (our study had a smaller sample size) and geographic location between the study sites may explain why these species were not isolated in our study. According to Wondimagegnehu et al. [21], agroecological factors contribute to the prevalence of EHV-1, with rates nearly double in midland areas. Similarly, El Brini et al. [31] noted significant regional differences in EHV-1 seroprevalence, observing higher rates in regions with a dense equid population, active breeding, communal living, and engagement in horse-related competitive activities and transportation. Additionally, the prevalence of EHV-2 and EHV-5 infections varied with altitude, with a higher proportion of equids in midland areas testing positive for EHV-2 compared to highland regions, while EHV-5 was more prevalent among equids in highlands than those in midland areas [32]. The content of a diet may also influence the occurrence of EHV-1. Marenzoni et al. [33] revealed alterations in the serum levels of some trace elements between EHV-1 noninfected and infected horses. However, our findings partially align with those of Stasiak et al. [24], who found no instances of EHV-1 positivity among the tested horses.

## 5. Limitation of the Study

This study showed the first EHV-5 outbreak in donkeys in the study area but had several limitations. It analyzed only 10 samples due to reagent scarcity and no EHV-4 analysis due to reagent unavailability. Moreover, there was no detection of the agent in damaged tissue, for example, immunohistochemistry, which would confirm the damage produced by the detected virus, and there was an absence of EHV-5 isolation and sequencing, which would confirm the identified virus.

## 6. Conclusion

From the outbreak in the study area, only EHV-5 was detected. The virus was identified from donkeys that showed nervous signs, respiratory signs, a combination of nervous and respiratory signs, and a combination of abortion, respiratory, and nervous signs. EHV-1 and EHV-2 were not detected in any of the cases. More detailed epidemiological and molecular studies should be performed to better understand the dynamics of the outbreak and inform preventive measures. Additionally, further studies involving damaged tissue samples, such as lung tissue, virus isolation, qPCR, and sequence analysis, should be carried out to confirm what was detected by PCR. Devising strategies to prevent and minimize the spread and occurrence of EHV-5 infection is crucial.

## Figures and Tables

**Table 1:** Primers for amplification of specific regions of the genome of EHV-1, EHV-2, and EHV-5.

Virus type	Target genes	Primer	Sequence (5′ ⟶ 3′)	Amplified products	Reference
EHV-1	ORF30	Forward	5′-GCTACTTCTGAAAACGGAGGC-3′	466 bp	[17]
		Reverse	5′-TATCCTCAGACACGGCAACA-3′		
EHV-2	gB	Forward	5′-GCCAGTGTCTGCCAAGTTGATA-3′	444 bp	[18]
		Reverse	5′-ATACGATCACATCCAATCCC-3′		
EHV-5	gB	Forward	5′-ATGAACCTGACAGATGTGCC-3′	293 bp	[19]
			5′-CACGTTCACTATCACGTCGC-3′		

**Table 2:** Clinical data observed in 23 EHV affected donkeys.

Clinical signs	Number of equids demonstrating the clinical signs (%)
Fever	13 (56.52%)
Coughing	12 (52.17%)
Enlargement of the mandibular and retropharyngeal lymph nodes	10 (43.48%)
Anorexia	17 (73.91%)
Lacrimation	3 (13.04%)
Nasal discharge	11 (47.83%)
Edema on lower abdomen/limb	4 (17.39%)
Urinary incontinence	2 (8.69%)
Ataxia and paresis	5 (21.74%)
Paralysis	4 (17.39%)
Head tilt	2 (8.69%)
Recumbence and inability to rise	3 (13.04%)
Abortion	4 (17.39%)

**Table 3:** Clinical findings of EHV outbreak based on different variables/factors.

Characteristics		Frequency	Percentage (%)
Sex	Male	9	39.1
	Female	14	60.9

Age	<3 yrs old	4	17.4
	3–8 yrs old	14	60.9
	>8 yrs old	5	21.7

BSC	Poor	13	56.5
	Moderate	7	30.4
	Good	3	13.0

Housing	Barn	6	26.1
	Loose	14	73.9

Contact with other equids	Yes	23	100
	No	0	0

**Table 4:** Conventional PCR was performed on DNA extracted from nasal swab samples collected from equids.

S. no	Animal	Sex	Age (yeas)	District	BCS	EHV-1	EHV-2	EHV-5
1	Donkey	Female	<3	Takusa	Poor	−	−	−
2	Donkey	Female	3–8	Takusa	Poor	−	−	−
3	Donkey	Female	3–8	Takusa	Moderate	−	−	+
4	Donkey	Male	3–8	Takusa	Moderate	−	−	+
5	Donkey	Female	>8	Takusa	Poor	−	−	+
6	Mule	Female	3–8	Takusa	Good	−	−	+
7	Donkey	Female	3–8	Belesa	Poor	−	−	+
8	Donkey	Male	3–8	Belesa	Poor	−	−	+
9	Donkey	Male	<3	Belesa	Poor	−	−	+
10	Donkey	Male	>8	Belesa	Moderate	−	−	—

Details of animals and PCR results for equine herpesvirus type-1, type-2, and type-5 in nasal swab samples were interpreted.

## Data Availability

The datasets used and/or analyzed during the current study are available from the corresponding author upon a formal request.
